# New Appliances and Things Medical

**Published:** 1899-04-15

**Authors:** 


### NEW APPLIANCES AND THINGS MEDICAL.

[We shall be glad to receive, at our Office, 28 & 29, Southampton Street, Strand, London, W.C., from the manufacturers, specimens of all new preparations and appliances which may be brought out from time to time.]

### ASEPTIC CATHETER TRAY.

(MESSRS. REYNOLDS AND BRANSON, 13, BRIGGATE, LEEDS.)

THIS is a tray which has been made for Mr. Reginald H. Lucy, of Plymouth, for use in catheterisation. It is so shaped—narrow in front and broader behind—as to fit comfortably between the thighs, with the narrow end against the perineum, so that in washing out the bladder, drawing off the urine, or douching wounds, the bed linen is not soiled nor the skin wetted. The tray is made of enamelled metal, is a cleanly contrivance, and easily sterilized.

### IZAL VAPOURISER.

(NEWTON CHAMBERS AND CO., THORNCLIFFE, SHEFFIELD.)

THE above vapouriser is an inexpensive and simple contrivance for impregnating the atmosphere with izal in suspension. Izal oil, of which izal is an emulsion, has a very high boiling point, it does not therefore volatilise at the temperature of boiling water, but remains mechanically suspended in the water vapour. The atmosphere may therefore become heavily charged with a particulate antiseptic if the vapouriser be kept working for some hours. A patient in a room with this apparatus at work may be supposed to inhale into his lungs considerable quantities of the preparation. We believe that satisfactory results have been attained from its use in cases of whooping-cough, croup, or incipient phthisis, but a larger series of experiences are required before one can state definitely that it has a curative influence on those cases in which relief can best be expected from a direct germicidal medicament. Whether izal can really reach the alveoli of the lung, and more especially the usually affected alveoli of the apices, is highly problematical. The instillation into the atmosphere of such a mixture as is supplied with the izal vapouriser, containing, as its does, a volatile turpentine, must necessarily be soothing to a troublesome cough. But it remains for it to prove its value as a curative agent. The vapouriser is of simple structure—in appearance and structure not unlike the "pyramid night-light feeder." A night-light supplies the source of heat, and a small metal receiver holds the izal mixture. A fenestrated and rotating collar regulates the heat and rate of evaporisation. It would certainly be an improvement if the collar could be so constructed as to rotate with greater freedom; unless arranged beforehand, the contents of the receiver are in danger of being upset in the adjustment.

### NON-ALCOHOLIC WINE.

(FIRST SWISS NON-ALCOHOLIC WINE COMPANY, LIMITED. LONDON DEPOT: COSENZA AND CO., 95, WIGMORE STREET.)

THE introduction of this novel wine is an attempt to solve the difficult problem of temperance. The wine is guaranteed by the makers as free from alcohol, and antiseptic—it is further stated to be the natural juice of the grape. The wine certainly has a striking similarity in taste and "sting" to ordinary fermented liquors. There are two varieties, the "sparkling" and the "still," and each of them is decidedly agreeable of its kind. The exhilarating effect of the former is distinctly noticeable, and in those cases in which there is a craving for alcohol this new pseudo-alcoholic beverage should certainly be tried. We anticipate good results in a certain proportion of the cases.

